# Supplementary material for: Structure of the portal complex from Staphylococcus aureus pathogenicity island 1 transducing particles in situ and in solution
Source: bioRxiv. 2023 Sep 18:2023.09.18.557803. Preprint. [Version 1] doi: 10.1101/2023.09.18.557803 (PMC10541612; doi:10.1101/2023.09.18.557803)

bioRxiv preprint doi: https://doi.org/10.1101/2023.09.18.557803; this version posted September 18, 2023. The copyright holder for this preprint (which was not certified by peer review) is the author/funder, who has granted bioRxiv a license to display the preprint in perpetuity. It is made available under aCC-BY 4.0 International license.

SUPPLEMENTARY INFORMATION

Supplementary Figure S1. Gold standard Fourier Shell Correlation (FSC) as a function of resolution between two half-maps for each of the reconstructions. **(A)** Portal in solution, C13. **(B)** SaPI1 empty capsid, C1. **(C)** SaPI1 full capsid, C1. **(D)** SaPI1 full capsid, icosahedral. **(E)** In situ portal focused, full capsid, C12. Panels A-E were generated in RELION; for each, the green curve is for the unmasked map, blue is for the masked map, red is for the phase randomized masked maps, and black is the masked curve corrected for the contribution of the mask.. **(F)** FSC for the in situ portal–capsid focused reconstruction from the full capsid, C1, generated in cryoSPARC. The blue, green, purple and red curves are for no mask, loose mask, tight mask, and corrected FSC, respectively.

Supplementary Figure S2. CP interactions. **(A)** View of atomic model (ribbon representation), superimposed on the icosahedral reconstruction density (mesh), viewed down the icosahedral fivefold axis, showing the interaction of A subunit (red) A-domains in the pentamer. One of the missing loops is indicated (arrow). **(B)** View down the icosahedral twofold axis, showing the interaction of A-domains from subunits B (blue), C (green) and D (yellow) in the hexamer. **(C)** Interaction of CP subunits around the icosahedral threefold axis, showing the turret formed by the P loops from three C subunits, shown as a molecular surface. One C subunit is colored a darker shade of green for clarity. The N-arms are shown in ribbon representation and indicated by the arrows. Adjacent D subunits (yellow) are shown in ribbon representation. **(D)** Interaction of CP subunits around the quasi-threefold axis formed by A (red), B (blue) and D (yellow) subunits, shown as a molecular surface. N-arms (arrows) and adjacent subunits are shown in ribbon representation.

bioRxiv preprint doi: https://doi.org/10.1101/2023.09.18.557803; this version posted September 18, 2023. The copyright holder for this preprint (which was not certified by peer review) is the author/funder, who has granted bioRxiv a license to display the preprint in perpetuity. It is made available under aCC-BY 4.0 International license.

Supplementary Table S1. Data collection, processing and refinement statistics.

Structure	Portals in solution	SaPI1 full capsid, C1	SaPI1 empty capsid, C1	SaPI1 full capsid, I1	SaPI1 empty capsid, I1	PP *in situ* (full) C12	PP *in situ* (empty) C12	PP *in situ* (full) C1
**Data Collection & Processing**								
Microscope	FEI Titan Krios G1							
Camera	Gatan K3 (counting)							
Energy filter	Gatan Quantum GIF							
Image collection software	Leginon							
Slit width (eV)	20							
Voltage (kV)	300							
Micrographs collected	3,168	3,581						
Defocus range (µm)	0.7–2.0	0.7–2.0						
Total exposure ($e^-/Å^2$)	50.97	35.26						
Frames per movie	46	38						
Pixel size (Å/pix)	1.08	1.33						
Reconstruction software	RELION-3.1	RELION-4.0	RELION-4.0	RELION-4.0	RELION-4.0	RELION-4.0	RELION-4.0	RELION-4.0; CryoSPARC v4.2
Particles extracted	1,811,063	97,939				222,708	13,500	259,743
Final particles	96,308	55,513	13,734	55,513	13,734	13,411	7,423	59,457
Symmetry imposed	C12	C1	C1	I1	I1	C12	C12	C1
Map resolution (Å, $FSC_{0.143}$)	2.4	3.9	4.2	3.1	3.2	3.2	3.5	3.4
Accession (EMDB)								
**Atomic Model Building**								
Local refinement software	ISOLDE, *Coot*							
Global refinement software	Phenix.real_space_refine							
Validation software	Phenix.validation_cryoem							
Sharpening B factor ($Å^2$)	-78.8				-70.3	-71.6		-97.8
Refinement resolution (Å)	2.5				3.2	3.2	3.5	
Model composition:								
Chains	1				4	2	2	
Atoms	3,900				9,463	3,911	3,923	
Hydrogens	0				0	0	0	
Residues	476				1,186	486	478	
Waters	0				0	0	0	
Ligands	0				0	0	0	
Bonds (RMSD):								
Length (Å) (# > 4σ)	0.005 (0)				0.006 (0)	0.008 (0)	0.007 (0)	
Angles (°) (# > 4σ)	1.163 (0)				1.207 (0)	1.126 (0)	1.103 (0)	
MolProbity score	0.89				0.91	0.64	0.83	
Clash score	0.78				0.42	0.38	1.29	
Ramachandran plot (%):								
Favored	97.26				95.83	98.13	97.89	
Allowed	2.74				4.17	1.87	2.11	
Outliers	0				0	0	0	
Rama-Z (Ramachandran plot Z-score, RMSD):								
whole (N = 732)	-0.78 (0.34)				-1.14 (0.23)	-0.87 (0.33)	-1.87 (0.32)	
helix (N = 75)	-1.18 (0.29)				-2.82 (0.24)	-0.75 (0.31)	-1.40 (0.29)	
sheet (N = 303)	1.23 (0.52)				0.81 (0.29)	0.58 (0.49)	-0.02 (0.56)	
loop (N = 354)	-0.28 (0.42)				-0.44 (0.25)	-0.75 (0.40)	-1.46 (0.34)	
Rotamer outliers (%)	0.23				0	0	0	
C_β outliers (%)	0				0	0	0	
Peptide plane (%):								
Cis proline/general	0.0/0.0				7.84	0.0/0.0	0.0/0.0	
Twisted proline/general	0.0/0.0				0	0.0/0.0	0.0/0.0	
CaBLAM outliers (%)	0.21				0.86	0.63	1.06	
Model fit vs. full map:								
$FSC_{0.5}$ ($FSC_{0.143}$)	2.5 (2.3)				3.33 (3.06)	3.4 (2.8)	3.7 (3.1)	
CC_{box}	0.34				0.4867	0.28	0.34	
CC_{mask}	0.84				0.8642	0.82	0.84	
Accession (PDB)								

bioRxiv preprint doi: https://doi.org/10.1101/2023.09.18.557803; this version posted September 18, 2023. The copyright holder for this preprint (which was not certified by peer review) is the author/funder, who has granted bioRxiv a license to display the preprint in perpetuity. It is made available under aCC-BY 4.0 International license.

Supplementary Table S2. Root-mean-square deviation (RMSD) between Cα atoms of portal proteins from different bacteriophages.

Structure (PDB ID)	80α PP solution		80α PP in situ, full	
	RMSD (Å)	Residues§	RMSD	Residues
80α in situ, full	0.59	320	–	–
	7.30	461	–	–
80α in situ, empty	0.57	319	0.45	453
	7.34	461	0.68	466
SPP1 solution (2JES)	1.25	170	1.10	178
	8.18	354	8.57	358
SPP1 in situ (7Z4W)	1.18	195	1.16	224
	9.40	417	7.24	422
P22 in situ, virion (3LJ5)	1.34	24	1.36	24
	17.89	392	16.08	389
Andhra in situ, virion (8EGR)	1.33	18	1.04	35
	20.71	289	26.04	268

§The RMSD values are calculated from the number of Cα atom pairs indicated. The first line corresponds to the pruned atom pairs (UCSF Chimera MatchMaker algorithm); the second line corresponds to all atom pairs in the sequences.

bioRxiv preprint doi: https://doi.org/10.1101/2023.09.18.557803; this version posted September 18, 2023. The copyright holder for this preprint (which was not certified by peer review) is the author/funder, who has granted bioRxiv a license to display the preprint in perpetuity. It is made available under aCC-BY 4.0 International license.

Figure S1

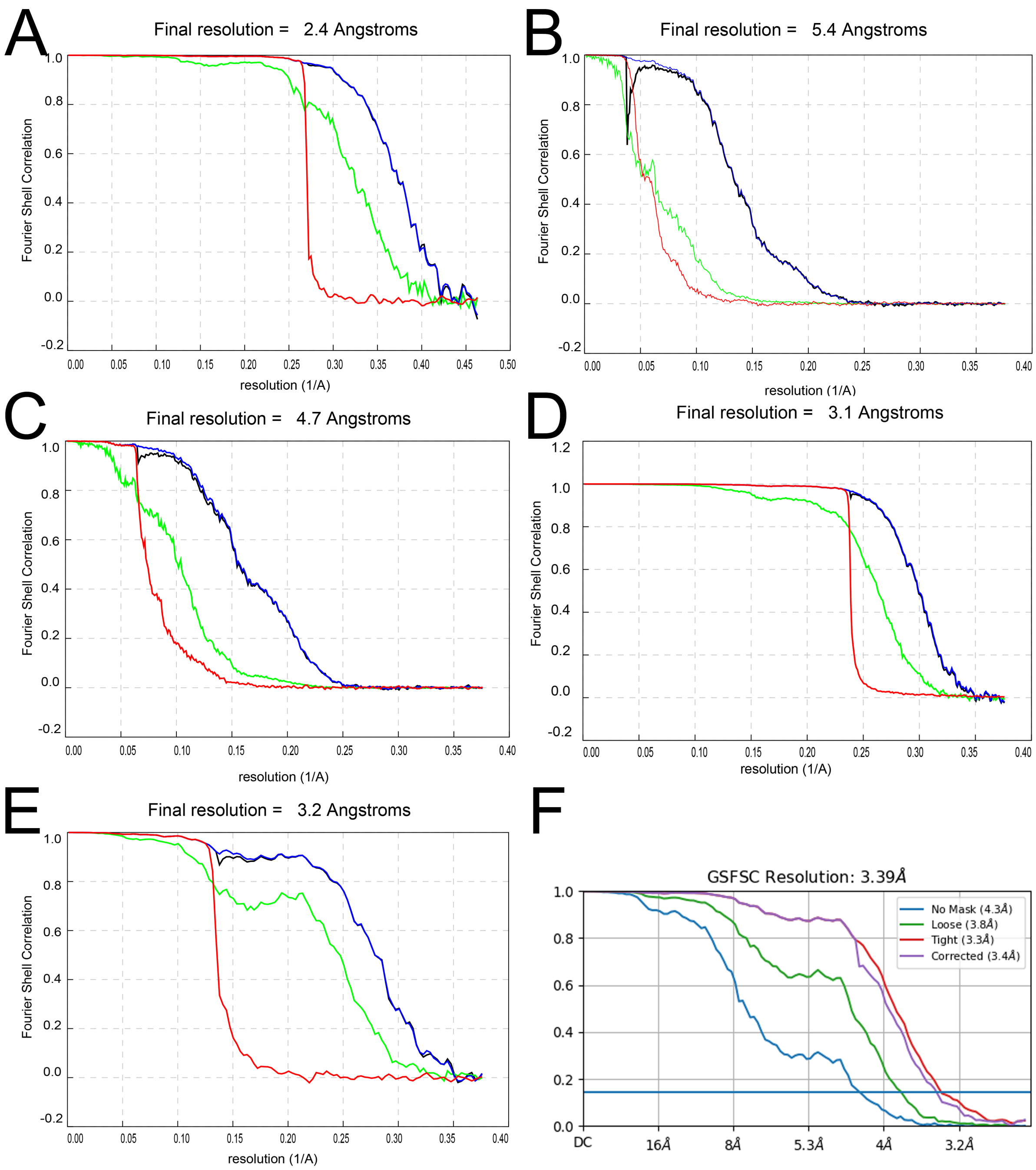

bioRxiv preprint doi: https://doi.org/10.1101/2023.09.18.557803; this version posted September 18, 2023. The copyright holder for this preprint (which was not certified by peer review) is the author/funder, who has granted bioRxiv a license to display the preprint in perpetuity. It is made available under aCC-BY 4.0 International license.

Figure S2

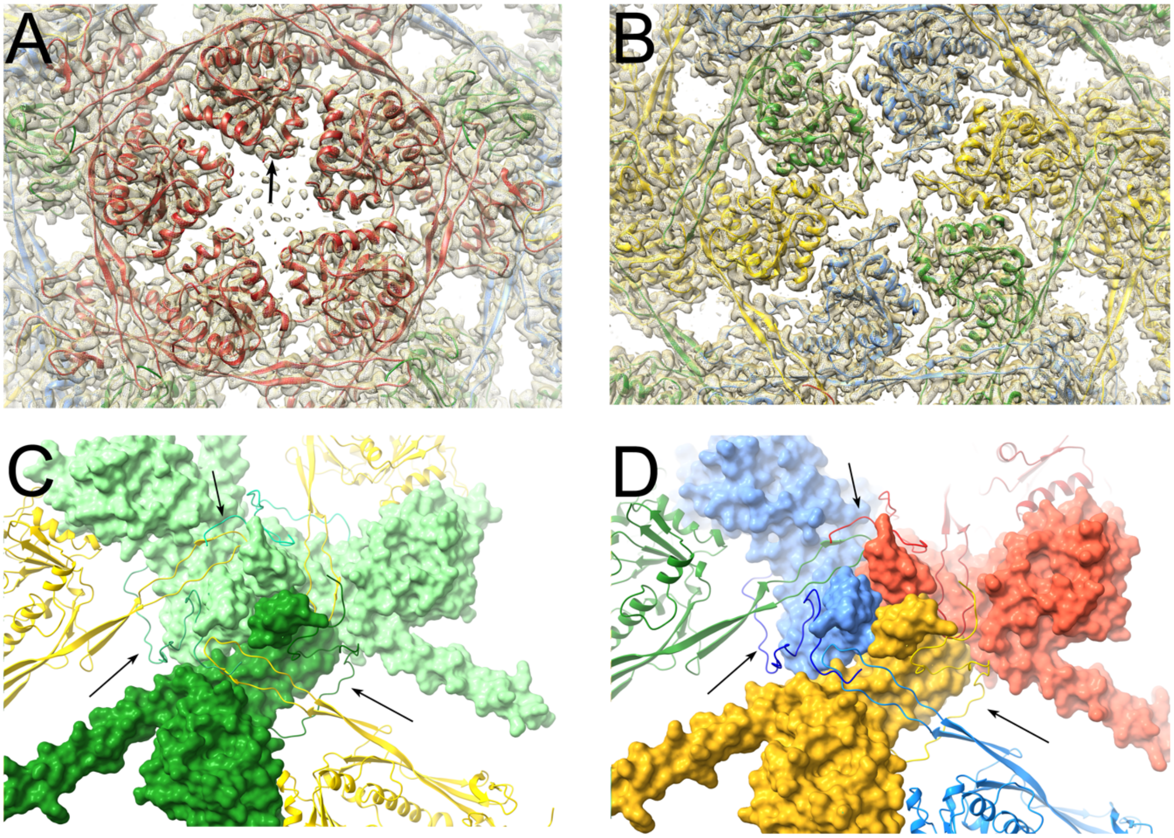

Supplement: Supplementary file 1 [file NIHPP2023.09.18.557803V1-supplement-1.pdf]
